# Kinetics of heterochiral strand displacement from PNA–DNA heteroduplexes

**DOI:** 10.1093/nar/gkab499

**Published:** 2021-06-14

**Authors:** Nandini Kundu, Brian E Young, Jonathan T Sczepanski

**Affiliations:** Department of Chemistry, Texas A&M University, College Station, TX 77843, USA; Department of Chemistry, Texas A&M University, College Station, TX 77843, USA; Department of Chemistry, Texas A&M University, College Station, TX 77843, USA

## Abstract

Dynamic DNA nanodevices represent powerful tools for the interrogation and manipulation of biological systems. Yet, implementation remains challenging due to nuclease degradation and other cellular factors. Use of l-DNA, the nuclease resistant enantiomer of native d-DNA, provides a promising solution. On this basis, we recently developed a strand displacement methodology, referred to as ‘heterochiral’ strand displacement, that enables robust l-DNA nanodevices to be sequence-specifically interfaced with endogenous d-nucleic acids. However, the underlying reaction – strand displacement from PNA–DNA heteroduplexes – remains poorly characterized, limiting design capabilities. Herein, we characterize the kinetics of strand displacement from PNA–DNA heteroduplexes and show that reaction rates can be predictably tuned based on several common design parameters, including toehold length and mismatches. Moreover, we investigate the impact of nucleic acid stereochemistry on reaction kinetics and thermodynamics, revealing important insights into the biophysical mechanisms of heterochiral strand displacement. Importantly, we show that strand displacement from PNA–DNA heteroduplexes is compatible with RNA inputs, the most common nucleic acid target for intracellular applications. Overall, this work greatly improves the understanding of heterochiral strand displacement reactions and will be useful in the rational design and optimization of l-DNA nanodevices that operate at the interface with biology.

## INTRODUCTION

**Figure 1.**
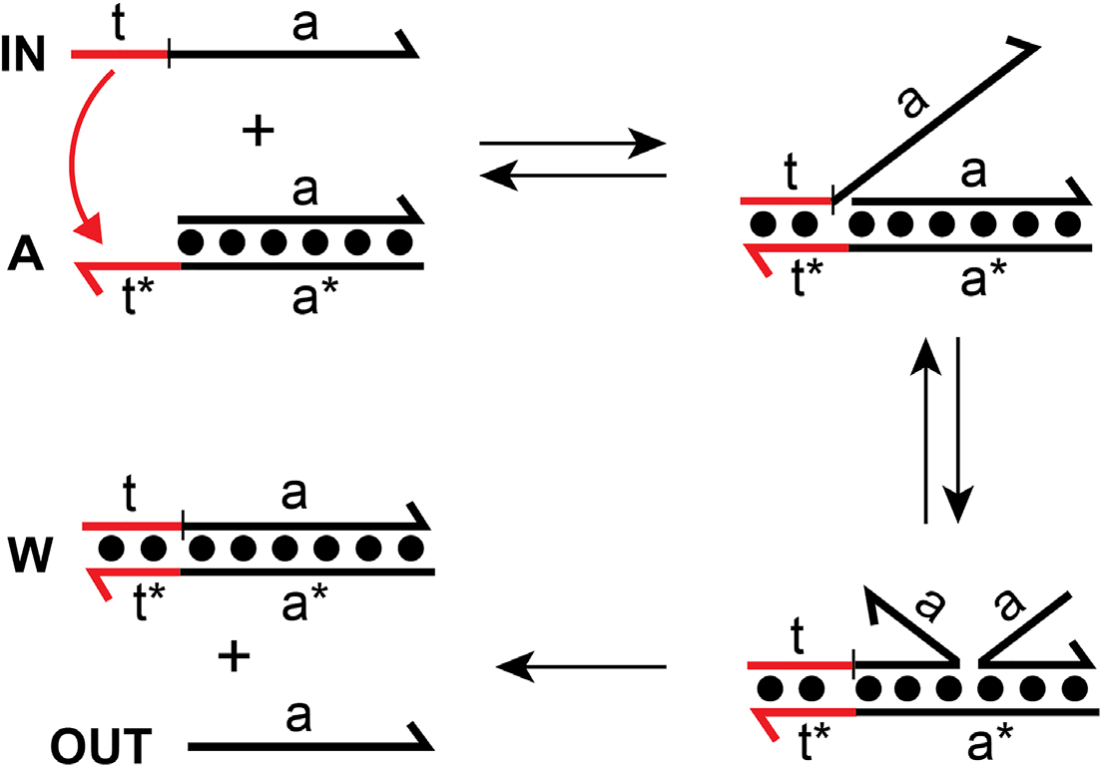
Toehold-mediated strand displacement reaction. DNA is depicted as lines with the half arrow indicating the 3′ end throughout the text. A substrate strand consisting of a single-stranded toehold domain (t*) and a branch migration domain (a*) is initially hybridized to an incumbent strand (OUT) to form duplex A. The input (or invader) strand IN is complementary to both the toehold (t) and branch migration domains (A) of the substrate strand. Displacement is initiated by binding of IN to the toehold (via t/t*) followed by a three-way branch migration process in which base pairs between the incumbent and substrate strands dissociate and are replaced by base pairs with invader IN. The reaction is complete once the incumbent strand (OUT) is fully displaced from duplex A.

Rationally engineered, DNA-based molecular devices with reconfigurable parts constitute the core of dynamic DNA nanotechnology. By design, the different modules of these devices exist in a state of non-equilibrium. Upon perturbation by specific molecular signals, the modules interact with each other and their environment via programmed Watson-Crick (WC) base-pairing interactions. In particular, a molecular mechanism referred to as toehold-mediated strand displacement underlies the operation of most dynamic DNA-based devices reported to date (Figure 1) (1,2). Over two decades of research have established fundamental mechanisms and design principles for DNA strand displacement systems that has fueled the development of an impressive repertoire of molecular devices with complex functionalities, including motor activity (3–6), structural reconfiguration (7–10), Boolean logic computation (11,12), spatiotemporal signal resolution (13) and enzyme-free catalytic amplification (14,15).

Applications at the interface with biology represent the primary motivation behind the development of many dynamic DNA nanodevices. By being constructed of DNA, RNA or related analogues, these devices are inherently compatible with cellular nucleic acids through WC base pairing interactions, thereby facilitating the interception and/or manipulation of molecular information in living systems. Use of DNA nanodevices for molecular sensing, imaging, and analysis of physiologically relevant biomarkers in fixed cells or tissues (16–18), surface of cell membranes (19,20), and even inside mammalian cells (21–23) has already been reported, representing significant progress in this direction. Yet, implementing DNA-based nanodevices within biological environments, and in particular live cells, remains an ambitious undertaking. Exogenously delivered DNA has a cellular half-life on the order of minutes and is susceptible to unintended interactions with endogenous macromolecules (24), all of which adversely affect the performance of the device. While use of chemical modifications, such as 2′-*O*-methyl ribonucleotides (25,26), locked nucleic acids (25) and phosphorothioate linkages (26) can confer nuclease stability, they also alter duplex thermostability (27) and hybridization kinetics (28,29) in a unpredictable manner. Indeed, compared to native DNA, strand displacement reactions involving chemically modified nucleic acids are poorly characterized, making the design of corresponding devices extremely challenging (22). Additionally, modified nucleotides can be toxic and tend to have adverse effects on cell viability (30,31). Due to these issues, modification-independent approaches for improving stability and retaining native hybridization parameters have also been explored, including the ligation of vulnerable free DNA ends and the use of more robust DNA architectures (32–34). However, these approaches have found only limited success. Importantly, none of the above approaches address potential off-target interactions of DNA-based devices with abundant cellular nucleic acids or other macromolecules, which further erode performance. Thus, there remains a need for new strategies aimed at improving the performance and reliability of DNA strand displacement systems within harsh biological environments.

l-DNA and l-RNA, the enantiomers (i.e. mirror-images) of native d-nucleic acids, have recently emerged as promising alternatives to chemical modification for the development of biocompatible nucleic acid-based technologies (35). Due to the inverted stereochemistry of the (deoxy)ribose moiety, l-oligonucleotides are mostly orthogonal to the stereospecific environment of natural biology. Consequently, l-DNA and l-RNA are highly resistant to nuclease degradation and less susceptible to non-specific interactions with other proteins and cellular macromolecules (36–38). They also avoid off-target hybridization with abundant cellular nucleic acids because oligonucleotides of opposite chirality (d versus l) are incapable of forming contiguous WC base pairs with each other (39–41). Importantly, as enantiomers, d- and l-nucleic acids have the same physical properties, including solubility, hybridization kinetics, and duplex thermal stability, making them identical from a design perspective (36,37,42). Based on these characteristics, use of l-nucleic acids as alternative materials for constructing strand displacement systems circumvents many of the drawbacks associated with implementing this technology in biological matrices.

**Figure 2.**
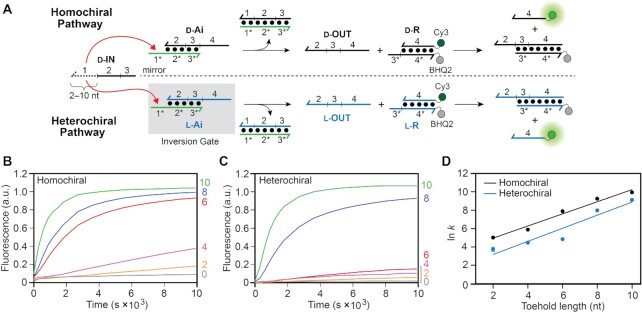
Strand displacement from a PNA–DNA heteroduplex is dependent on toehold length and stereochemistry. (**A**) Schematic illustration of the strand displacement reaction system depicting both the homo- and heterochiral pathways. d-DNA is shown in black, l-DNA is shown in blue, and PNA is shown in green throughout the text. The half-arrow denotes the C-terminus of the PNA strand. The sequences of all strands are depicted in Supplementary Figure S1 and Supplementary Table S1. (**B**, **C**) Fluorescence monitoring (Cy3) of the homochiral (B) and heterochiral (C) reaction pathways initiated with inputs (d-IN) having toehold domains varying in length from 0–10 nucleotides (nt). The length of the toehold is indicated on the right y-axis. The reactions depicted contained 150 nM d-IN, 100 nM d/l-Ai, 300 nM d/l-R, 300 mM NaCl, 1 mM EDTA, and 10 mM Tris (pH 7.6) and were carried out at 37°C. Fluorescence in all figures is reported in units such that 1.0 is the fluorescence of the maximally activated reporter control and 0.0 is the background of the quenched reporter complex (d/l-R). (**D**) Semilogarithmic plot showing the exponential dependence of calculated rate constants on toehold length. All calculated rate constants are listed in Supplementary Table S2.

Inspired by this idea, we recently developed a toehold-mediated strand displacement methodology for transferring sequence information between otherwise orthogonal oligonucleotide enantiomers (43). Our approach, termed ‘heterochiral’ strand displacement, relies on a heteroduplex between a *chiral* strand of l-DNA and an *achiral* strand of peptide nucleic acid (PNA), which hybridizes to DNA/RNA irrespective of chirality (Figure 2A). We refer to this complex (l-Ai) as the ‘inversion gate’. During the reaction, the d-nucleic acid input strand (d-IN) hybridizes to the inversion gate via the achiral toehold domain (1*) on the PNA strand, leading to the displacement of the incumbent l-DNA strand (l-OUT) in the process. In this way, the sequence information within the d-input, and specifically domains 2 and 3, is ‘inverted’ into l-DNA. In principle, the inversion gate allows for any d-nucleic acid input signal to be sequence-specifically interfaced with a robust nanodevice composed of bio-orthogonal l-DNA/RNA. For example, this approach has been used to interface microRNAs with l-DNA-based logic circuits and catalytic amplifiers *in vitro* (43,44), and with an l-RNA-based fluorescent biosensor in live cells (45).

Although heterochiral strand displacement systems have shown remarkable promise, the key reaction—strand displacement from a PNA–DNA heteroduplex—remains poorly characterized, potentially limiting design capabilities. Indeed, a detailed understanding of all-DNA strand displacement kinetics and underlying biophysical mechanisms has greatly aided the rational design of dynamic DNA nanodevices having diverse behaviors. In the same way, establishment of a well-understood kinetic model of strand displacement from PNA–DNA heteroduplexes will be important for the rational design and optimization of dynamic l-DNA/RNA nanodevices that can be reliably interfaced with native biology. To this end, we determined the impact of several common design parameters, including toehold length and mismatches, on the kinetics of strand displacement from PNA–DNA heteroduplexes. To better understand how stereochemistry contributes to reaction kinetics, we directly compared homo- and heterochiral reaction pathways (Figure 2A), wherein the input strand (d-IN) has the same or opposite stereochemistry as the PNA–DNA heteroduplex d-Ai and l-Ai, respectively. We show that the rate of strand-displacement from PNA–DNA heteroduplexes can be tuned across several orders of magnitude based on the length of the PNA toehold, mismatch position, and stereochemical configuration of the reaction. Notably, heterochiral strand displacement reactions are slower than their homochiral equivalent, despite the overall change in free energy being identical. We experimentally investigate the source of this intriguing kinetic penalty and demonstrate how stereochemistry can be used to control strand displacement kinetics. Despite having a slower rate, heterochiral strand displacement is highly sensitive to mismatches on the input strand, especially within the toehold domain, providing a potential advantage for engineering nucleic acid-based probes. Furthermore, heterochiral strand displacement rates are substantially enhanced when using RNA inputs, which represent the most common type of nucleic acid target for biosensing applications. Overall, this work establishes a basic set of design considerations to guide the future development of robust heterochiral strand displacement systems, thereby broadening the scope and applicability of l-DNA/RNA nanodevices for practical biomedical applications.

## MATERIALS AND METHODS

### Materials

Oligonucleotide synthesis reagents, d-nucleoside phosphoramidites, 6-Fluorescein phosphoramidite (6-FAM) and the Cyanine 3 (Cy3) phosphoramidite were purchased from Glen Research (Sterling, VA). l-Nucleoside phosphoramidites were purchased from ChemGenes (Wilmington, MA). Black Hole Quencher 2 (BHQ2) CPG resins were purchased from LGC Biosearch Technologies (Petaluma, CA). Peptide nucleic acids (PNAs) were purchased from Panagene (Daejeon, South Korea) at 99.9% purity and were not purified further. All other reagents were purchased from Sigma Aldrich (St. Louis, MO).

### Oligonucleotide synthesis and purification

Unmodified d-oligonucleotides were purchased from Integrated DNA Technologies (Coralville, IA), and all l-oligonucleotides were synthesized in house using an Expedite 8909 DNA/RNA synthesizer. Terminal labeling of the 5′ end with either Cy3 or 6-carboxyfluorescein (6-FAM) was carried out using the corresponding phosphoramidites, which were coupled based on the manufacturers recommended protocols. All 3′-BHQ2 modified oligonucleotides were obtained by conducting the synthesis on the corresponding BHQ2 CPG resin. Following synthesis and deprotection, single-stranded oligonucleotides were purified by 20% denaturing polyacrylamide gel electrophoresis (PAGE; 19:1 acrylamide: bisacrylamide). Purified oligonucleotides were excised from the gel and eluted overnight at room temperature in a buffer consisting of 200 mM NaCl, 10 mM EDTA, and 10 mM Tris (pH 7.6). The solution was filtered to remove gel fragments, then desalted by ethanol precipitation. The obtained pellet was resuspended in water and quantified by measuring the absorbance at 260 nm using a Nanodrop 2000c spectrophotometer (Thermo Fisher Scientific, Waltham, MA). Commercial PNAs, obtained as lyophilized solids, were reconstituted at 100 μM in water and used without further purification. Individual strands were quantified using the extinction coefficients provided by the manufacturer.

### Preparation and characterization of duplex reaction components

In order to form complexes d/l-Ai, d/l-R and d-Aq the corresponding oligonucleotides (Supplementary Figure S1 and Supplementary Table S1) were annealed in a reaction mixture containing the appropriate amount of each strand (see below), 300 mM NaCl, 1 mM EDTA, 10 mM Tris (pH 7.6) and were heated to 90°C for 3 min then cooled slowly to room temperature over 1 h. For d/l-Ai, 50 μM of PNA_L_ was annealed to 75 μM d/l-OUT. For d/l-R, 100 μM d/l-F was annealed to 150 μM d/l-Q. For d-Aq, 10 μM of PNA_S_ was annealed to 10.5 μM d-OUT_Q_. Whereas d-Aq was used directly, complexes d/l-Ai and d/l-R were further purified by 20% native PAGE (19:1 acrylamide:bisacrylamide) and single bands were carefully excised from the gel. The gel fragments were crushed and eluted overnight at room temperature in the same buffer that was used for annealing, and the suspension was filtered through a 0.2 μm filter. The concentration was estimated from UV absorbance at 260 nm using the combined extinction coefficients of the individual strands comprising the duplex.

For a more accurate determination of the concentration of duplex components d/l-Ai and d/l-R, a calibration curve of Cy3 fluorescence of free strand d/l-F was generated over a range of concentrations from 100 nM to 300 nM, as measured at excitation/emission wavelength of 520 nm/580–640 nm (bandpass filter). These measurements were taken at 37°C in a buffer containing 300 mM NaCl, 1 mM EDTA, 10 mM Tris (pH 7.6). This calibration curve (i.e. the linear relationship between the concentration of strand d/l-F and Cy3 fluorescence) was then used to determine the concentration of components d/l-Ai and d/l-R based on Cy3 fluorescence following a strand displacement reaction under the same conditions. For example, the concentration of d/l-R was determined by first reacting 100 nM of d/l-R (estimated) with a large excess of d/l-OUT in order to drive the strand displacement reaction to completion. The Cy3 signal generated from this reaction was then compared to the calibration curve to determine the amount of strand d/l-F present in d/l-R, and thus, its concentration. Prior to use, fresh dilutions of each complex were prepared in the presence of 10 μM poly[T] carrier oligonucleotide to prevent loss of material from sticking to plastic surfaces of tubes and pipette tips (46).

### Monitoring strand displacement reactions by fluorimetry

Strand displacement reactions were monitored using a Glomax Discover multi-well plate reader (Promega Corp.) using excitation/emission wavelengths 520 nm/580–640 nm (bandpass filter for Cy3). Reaction mixtures contained either 60 nM or 300 nM d/l-R, either 30 nM or 150 nM of the indicated input strand, 300 mM NaCl, 1 mM EDTA, and 10 mM Tris (pH 7.6) and were initiated by the addition of either 20 nM or 100 nM of d/l-Ai, respectively. The negative control contained no input. Each experiment was run side-by-side with a ‘pre-activated’ reaction mixture that contained 1 μM of d/l-IN_TH_10 (Supplementary Table S1), either 20 nM or 100 nM d/l-Ai, either 60 nM or 300 nM d/l-R, 300 mM NaCl, 1 mM EDTA and 10 mM Tris (pH 7.6). Pre-activated reaction mixtures were incubated for 10 minutes prior to use in order to fully activate the reporter complex. Each reaction mixture was in a final volume of 30 μL and carried out at 37°C unless otherwise specified.

All strand displacement reactions were normalized to the signal from the pre-activated reaction representing the maximum achievable fluorescence using Equation (1):

(1) $$\begin{equation*}{F_n} = \frac{{\;F - {F_0}}}{{{F_c} - {F_0}}}\;\end{equation*}$$

where *F_n_* is the normalized fluorescence intensity, *F* is the measured fluorescence, *F*_0_ is the fluorescence of the quenched reporter complex and *F*_c_ is the fluorescence of the activated reporter complex at the time a measurement was taken. Because the PNA–DNA heteroduplex (d/l-Ai) is the limiting reagent in all cases, 1.0 normalized fluorescence units (FU) corresponds to either 20 or 100 nM of activated reporter d/l-R (i.e. free strand d/l-F).

### Rate constant fitting procedure

All strand displacement reactions performed for this study are assumed to be second-order reactions with respect to the input strand and PNA–DNA heteroduplex Ai as described previously (43,46,47). As indicated above, two concentration regimes of strand displacement components were used to extract rate constants: (A) 150 nM d-IN, 100 nM d/l-Ai and 300 nM d/l-R and (B) 30 nM d-IN, 20 nM d/l-Ai and 60 nM d/l-R.

Very slow strand displacement reactions that are unlikely to go to completion, such as short heterochiral toeholds (2–4 nt), and certain mismatches, were fit using Equation (2) (47):

(2) $$\begin{equation*}{\rm ln}\;\frac{{\left( {{{\left[ {{\rm{Ai}}} \right]}_0} - \left[ {{\rm{OUT}}} \right]} \right){{\left[ {{\rm{IN}}} \right]}_0}}}{{\left( {{{\left[ {{\rm{IN}}} \right]}_0} - \left[ {{\rm{OUT}}} \right]} \right){{\left[ {{\rm{Ai}}} \right]}_0}}} = \;\left( {{{\left[ {{\rm{Ai}}} \right]}_0} - {{\left[ {{\rm{IN}}} \right]}_0}} \right)kt\end{equation*}$$

where [OUT] is the concentration of displaced d/l-OUT at time *t*, and [IN]_0_ and [Ai]_0_ are the initial concentrations of the input strand and the PNA–DNA heteroduplex, respectively. The concentration of displaced incumbent strand [OUT] at any given time *t* was obtained by multiplying the respective normalized fluorescence intensity by [Ai]_0_. The left-hand side of the Equation (2) was plotted versus *t*, and the respective rate constant (*k*) was extracted from the slope of the linear fit to the plot (Supplementary Figure S2; Representative fits in the SI). We note that minor deviations from second-order kinetics were observed for some very slow reactions, and in particular, those resulting from short toeholds in the heterochiral configuration (Supplementary Figure S2b). This is not unexpected (48). However, for the sake of a uniform analysis of reaction rates, we have reported all rate constants as second-order.

Fast reactions were fit using Equation (3), which is rearranged from Equation (2):

(3) $$\begin{equation*}{\left[ {{\rm{OUT}}} \right]_n} = \frac{{{{\left[ {{\rm{IN}}} \right]}_0}\left( {1 - ex{p^{[kt\left( {{{\left[ {{\rm{Ai}}} \right]}_0} - {{\left[ {{\rm{IN}}} \right]}_{0)}}} \right]}}} \right)}}{{{{\left[ {{\rm{IN}}} \right]}_0} - {{\left[ {{\rm{Ai}}} \right]}_0}ex{p^{\left[ {kt\left( {{{\left[ {{\rm{Ai}}} \right]}_0} - {{\left[ {{\rm{IN}}} \right]}_0}} \right)} \right]}}}}\;\end{equation*}$$

where [OUT]_*n*_ is the normalized fluorescent intensity at time *t*, [IN]_0_ and [Ai]_0_ are the initial concentrations of the input strand and the PNA–DNA heteroduplex respectively. To extract rate constants (*k*), Equation (3) was fit to all the data points.

All rate constants are reported as the mean value of at least three replicates, and the corresponding standard deviation has been used as a measure of the error. The data was plotted in Graphpad Prism and the extracted rate constants are listed in Supplementary Table S2 for all strand displacement reactions reported herein.

### Melting temperature analysis

Melting experiments were performed on a Bio-Rad CFX96 Touch Real-Time PCR instrument and fluorescence was measured using excitation/emission wavelengths of 520 nm/580–640 nm (bandpass filter for Cy3). Reaction mixtures contained an equimolar ratio of either d- or l-IN_F_ and d-Aq within a buffer comprising 300 mM NaCl, 1 mM EDTA, and 10 mM Tris (pH 7.6). d/l-IN_F_ lacks the branch migration domains found in d-IN (domains 2 and 3), instead containing five dT residues and a 5′-6-FAM dye (Supplementary Figure S1 and Supplementary Table S1). The incumbent strand (d-OUT_Q_) within heteroduplex d-Aq was labeled internally with a quencher (BHQ2) such that binding of either d- or l-IN_F_ to the toehold domain (1*) resulted in fluorescence quenching. d/l-IN_F_ and d-Aq were annealed at 6 concentrations (*C*_t_) of 0.5, 1, 2, 3, 4 and 6 μM, where *C*_t_ represents the combined concentration of d/l-IN_F_ and d-A_q_. All reactions also contained 10 μM poly[T] carrier oligonucleotide to prevent loss of material from sticking to plastic surfaces of tubes and pipette tips (46). Individual reaction mixtures containing the indicated concentrations of either d- or l-IN_F_ and d-Aq were incubated at 10°C for 10 min then heated to 70°C in 2°C increments with an equilibration time of 5 min for each step. Each melting reaction was run side-by-side with a positive control containing only d-IN_F_ or l-IN_F_ under identical conditions. Fluorescence readings were taken at the end of each equilibration period, prior to the next 2°C temperature increase. The 70°C maximum temperature is ∼20°C above the predicted melting temperature of the toehold duplex (1/1*) (49).

To construct a temperature dependent melting profile at each concentration, the measured fluorescence signal was first corrected for (1) background and (2) temperature-dependent changes in fluorescence using Equation (4):

(4) $$\begin{equation*}{{{F}}_{{\rm{corr}}}} = {\rm{\;}}\frac{{{{{F}}_{{\rm{melt}}}} - {{{F}}_{{\rm{bkgnd}}}}}}{{{{{F}}_{{\rm{pos\;}}}} - {{{F}}_{{\rm{bkgnd}}}}}}\end{equation*}$$

where *F*_corr_ is the corrected fluorescence, *F*_melt_ is the measured fluorescence of the melting reaction (d- or l-IN_F_ and d-Aq), *F*_pos_ is the measured fluorescence of the positive control (d- or l-IN_F_ only) and *F*_bkgnd_ is the measured background fluorescence of an empty well. *F*_corr_ was plotted against temperature to derive the melting profile. The first and second derivatives of these curves were approximated in Microsoft Excel, and the value at which the second derivative intersected the x-axis was considered the melting temperature for the corresponding concentration (*C*_t_). The inverse of the melting temperature (1/*T*_m_) was plotted against ln *C*_t_ (Supplementary Figure S4), generating a line from which thermodynamic parameters Δ*H*° and Δ*S*° of toehold binding can be calculated according to Equation (5) (50,51):

(5) $$\begin{equation*}\frac{1}{{{T_{\rm m}}}} = \frac{{\left( {n - 1} \right)R}}{{\Delta H^\circ }}\;{\rm ln}{C_T} + \frac{{\left[ {\Delta S^\circ - \left( {n - 1} \right)R{\rm ln}\,2n} \right]}}{{\Delta H^\circ }}\end{equation*}$$

where *T*_m_ is the melting temperature, *C*_t_ is the combined concentration of d/l-IN_F_ and d-Aq, *n* is the molecularity of the binding reaction (assumed to be *n* = 2 for d/l-IN_F_ + d-Aq) and ΔH° and ΔS° are the enthalpy and entropy changes associated with the toehold binding event, respectively.

## RESULTS AND DISCUSSION

### Toehold length and stereochemistry modulate the kinetics of strand displacement from a PNA–DNA heteroduplex

Toehold-mediated strand displacement reactions are initiated by binding of the input strand (also referred to as an ‘invader’) to the toehold domain. Toeholds increase the rate of strand displacement by increasing the probability that the incumbent strand is successfully replaced by the input strand once bound. As a result, the overall rate of the reaction, which can be approximated as being second order, is strongly dependent on toehold stability (46,52). For an all-DNA system, the rate of strand displacement can be adjusted over 6 orders of magnitude by simply changing the length and sequence of the toehold (46,53). Thus, the toeholds represent a key design parameter for kinetic control over engineering dynamic DNA devices.

To investigate the effects of toehold length on strand displacement from a PNA–DNA heteroduplex (d/l-Ai), we utilized a reaction system originally reported by Zhang and Winfree (Figure 2A) (46). The sequences were chosen (and confirmed via NUPACK) (54) to have no secondary structure within single-stranded regions to ensure decoupling of the second-order process of strand displacement from the first order process of secondary structure unfolding. The reactions were monitored using an indirect reporter system in which the displaced incumbent strand (d/l-OUT) in turn displaces a fluorophore (Cy3)-labeled strand from the reporter duplex (d/l-R). This indirect strategy avoids the need to label primary reaction components, which could have unpredictable effects on their interactions (55,56). d-DNA inputs (d-IN) were used for all strand displacement reactions. Toehold length was varied by sequentially truncating the toehold domain (1) on the input strand from 10 to 0 nucleotides, whereas the length of the toehold domain (1*) on the PNA strand of the heteroduplex (d/l-Ai) remained constant at 10 nucleotides. The chirality of the incumbent strand within the PNA–DNA heteroduplex was either d-DNA (d-OUT; for the homochiral reaction pathway) or l-DNA (l-OUT; for the heterochiral reaction pathway), and the two reaction pathways were monitored separately using either d-R or l-R reporter complex, respectively (Figure 2a). Because our ultimate goal is to provide design principles for heterochiral strand displacement devices that are compatible with living systems, and in particular human cells, all reactions were carried out under simulated physiological conditions (300 mM NaCl, pH 7.6 and 37°C).

Figure 2 shows how the rate of both homo- and heterochiral strand displacement from a PNA–DNA heteroduplex depends on toehold length. For both reaction configurations, the rate dependency on toehold length is roughly exponential (Figure 2d), varying by up to two orders of magnitude for toeholds between 2 and 10 nucleotides long. Consistent with our previous observations (43), the homochiral reaction pathway is faster than the heterochiral reaction pathway for any given toehold length. Taking the 6-nt toehold as an example, the homochiral strand displacement reaction is more than an order of magnitude faster than its heterochiral counterpart, despite their sequences and overall change in free energy being identical. This observation implies that helical inversion of the PNA–DNA heteroduplex during heterochiral strand displacement imposes an additional kinetic barrier relative to the homochiral reaction configuration, which may be due, in part, to weaker toehold interaction between the d-DNA input strand and l-Ai. We directly address the contribution of toehold binding on the observed reaction kinetics below. We note that no appreciable reaction occurred in the absence of an input (Supplementary Figure S3).

Rate constants calculated for strand displacement from PNA–DNA heteroduplexes are considerably slower than for those previously reported for all-DNA reactions for the same toehold length, regardless of reaction configuration (Supplementary Table S2). Moreover, the rate fails to saturate for longer toeholds. For comparison, rate enhancement plateaus once the toehold becomes longer than ∼6-nucleotides for traditional all-DNA strand displacement reactions carried out at 23°C (46,52,53). We interpret these results as being partly due to the increased temperature at which our reactions were carried out (37°C) (57), and partly due to the greater enthalpic cost associated with disrupting PNA–DNA base pairs compared to DNA-DNA base pairs (58,59), which imposes a higher penalty for initiation and propagation of branch migration (52). Nevertheless, rate constants for longer PNA toeholds under these conditions still approach the lower bounds for what has been observed for all-DNA strand-displacement reactions (∼10^5^ M^−1^ s^−1^), with potential for further improvement. For example, the use of stronger toehold sequences (i.e. higher G/C content) and/or introduction of mismatches into the PNA–DNA heteroduplex (60) could be used to increase strand displacement kinetics without further increasing toehold length, and will be the subject of future investigations.

Collectively, these data indicate that the rate constant for strand displacement from a PNA–DNA heteroduplex can be predictably tuned by adjusting toehold length and reaction configuration. Importantly, PNA toeholds ≥ 8 nucleotides long provide reaction rates that are sufficiently fast for most *in vitro* and intracellular applications of heterochiral strand displacement. That being said, caution must be used when designing longer PNA toeholds in order to avoid undesirable secondary structures that impede binding to the input strand, as well as purine rich sequences that might promote aggregation (61).

### Strand displacement from PNA–DNA heteroduplexes is sensitive to mismatches

**Figure 3.**
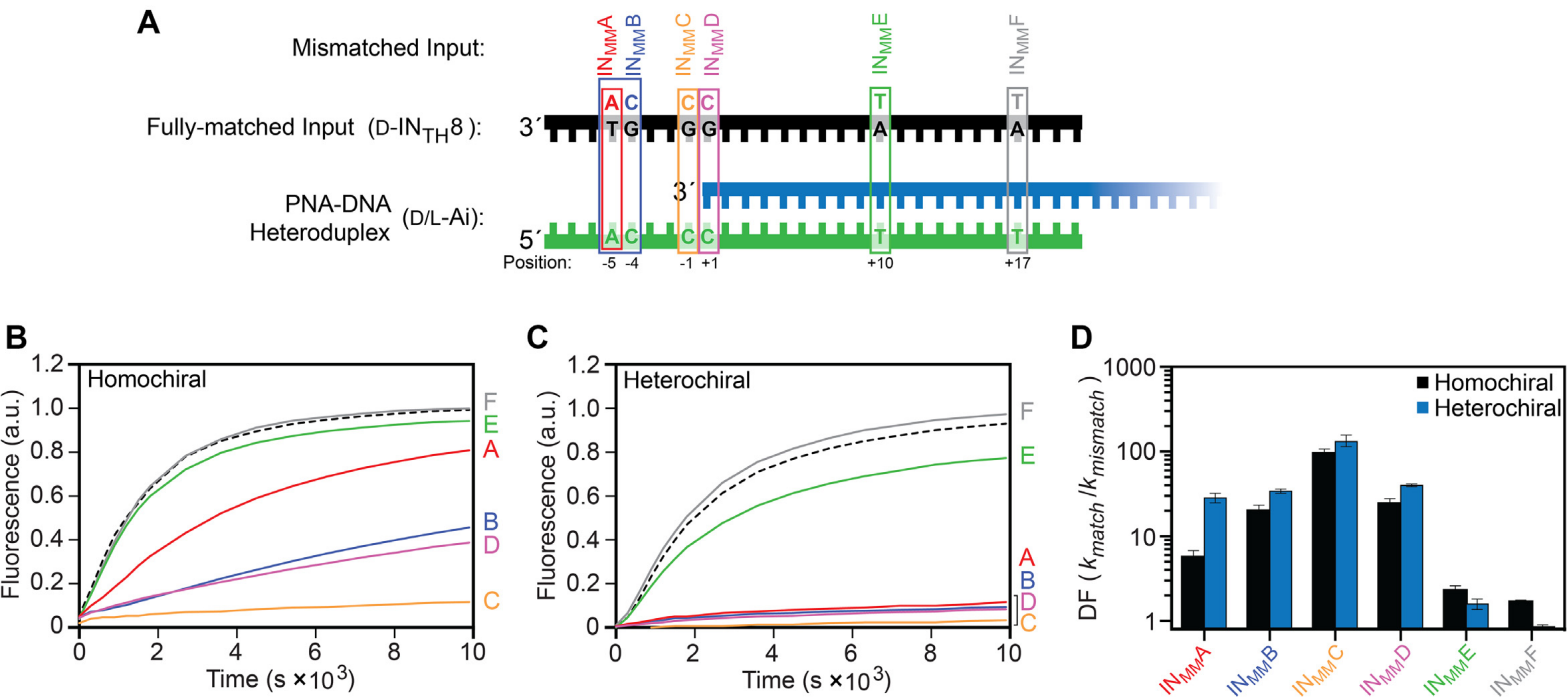
The position of a mismatch affects the rate of strand displacement from PNA–DNA heteroduplexes. (**A**) Schematic of the mismatched inputs used in this study. The identity of the mismatched nucleotide relative to the fully matched input (d-IN_TH_8) is shown above the strand. (B, C) Fluorescence monitoring (Cy3) of the homochiral (**B**) and heterochiral (**C**) reaction pathways initiated with different mismatched inputs in (A). The identity of the mismatched input (IN_MM_A–F) is indicated on the right y-axis. The reaction initiated with the fully-matched input (d-IN_TH_8) is shown as a black dotted line. Reactions depicted here were carried out as described in Figure 2. (**D**) Kinetic discrimination factors (DF = *k*_match_/*k*_mismatch_) of hetero- and homo-chiral reactions towards inputs having different mismatches, where *k*_match_ and *k*_mismatch_ are the calculated rate constants for a fully-matched and mismatched input, respectively. Error bars represent standard deviation from three independent experiments.

The rate of DNA strand displacement reactions can be modulated over several orders of magnitude by introducing one or more mismatches between the input strand and the target duplex (48,60). Rational positioning of mismatches provides a useful control mechanism for competitive reaction networks (60,62) and enables the design of strand displacement-based nucleic acid probes having a high-degree of mismatch discrimination (63,64). A common practical application is the detection of single nucleotide polymorphisms (SNPs), which are of great diagnostic value (65–67). Given the design capabilities enabled by the incorporation of mismatches into all-DNA strand displacement systems, we sought to characterize the influence of mismatches on the rate of homo- and heterochiral strand displacement from a PNA–DNA heteroduplex. A series of inputs containing mismatches at a single position (and one input containing two mismatches within the toehold binding domain) were generated from input strand (d-IN_TH_8) having an 8-nucleotide toehold domain (Figure 3A). Placement of the mismatches were, in part, informed by previous literature on the effects of mismatches on rates of all-DNA strand displacement reactions, as well as PNA–DNA hybridization (48,58,62,63).

We first considered inputs containing mismatches within the toehold domain. Compared to the homochiral reaction pathway, heterochiral stand displacement from a PNA–DNA heteroduplex is much more sensitive to a single mismatch positioned near the middle of the toehold domain (IN_MM_A), both in terms of rate and equilibrium yield (i.e. fraction d/l-OUT displaced) (Figure 3B, C and Supplementary Table S2). This observation likely reflects an overall weaker toehold interaction between the input strand and the PNA–DNA heteroduplex of opposite chirality during the heterochiral reaction. The kinetic discrimination factors (DF) (DF = *k*_match_/*k*_mismatch_) for IN_MM_A are 5.8 and 29.4 for the homo- and heterochiral reactions, respectively (Figure 3D). For comparison, a DF of ∼2 has been reported for an all-DNA strand displacement system with a similarly positioned mismatch (63). This suggests that, regardless of the reaction configuration, strand displacement from PNA–DNA heteroduplexes is highly sensitive to single mismatches within the toehold domain. When two mismatches were present in the toehold (IN_MM_B), the DF for the homochiral reaction was greatly improved (DF = 20.8), but was still less than the heterochiral reaction (DF = 36.1) (Figure 3D). Interestingly, a mismatch positioned immediately before the branch migration domain (IN_MM_C; position −1) almost completely inhibits strand displacement for both homo- and heterochiral reaction pathways (DF > 100 for both reaction configurations). Computational studies suggest that coaxial stacking between the invading (input) and incumbent duplexes at the branch point (i.e. −1/+1) play an important role in the mechanism of branch migration initiation (52), as well as the kinetics of DNA hybridization (57). Therefore, a mismatch at position −1 (IN_MM_C) is expected to largely disrupt these interactions, greatly raising the activation barrier for initiating branch migration and reducing the overall reaction rate.

We next examined inputs containing mismatches within the branch migration domain (Figure 3A). For both homo- and heterochiral reaction pathways, introduction of a mismatch immediately adjacent to the toehold domain (+1; IN_MM_D) greatly impeded strand displacement (Figure 3B, C), which is consistent with all-DNA strand displacement reactions (48,56,62). While this mismatch does not compromise toehold stability, the system must enter an energetically less-favorable state to initiate branch migration because the input strand must immediately enclose a mismatch. This greatly reduces the probability that the input will successfully displace the incumbent strand prior to spontaneous detachment from the toehold, and thus, reduces the overall reaction rate. Again, the greater sensitivity of the heterochiral reaction configuration to this mismatch (IN_MM_D) is likely due to weaker toehold binding. Relative to IN_MM_D, mismatches within the middle or near the end of the branch migration domain (IN_MM_E and IN_MM_F) had only a modest effect on the rate of strand displacement for both reaction configurations (Figure 3D). Although inputs IN_MM_E and IN_MM_D must overcome similar energy barriers at the site of the mismatch, IN_MM_E has a more stable pre-mismatch state because it is able to form more base pairs with the PNA prior to encountering the mismatch. This increases the probability of successful displacement by IN_MM_E relative to IN_MM_D, leading to faster overall reaction kinetics and reduced mismatch discrimination. The inability of d/l-Ai to discriminate against IN_MM_F can be explained by an alternative dissociation pathway for release of the incumbent strand as the branch point approaches the end of the branch migration domain, wherein spontaneous melting of the remaining base pairs provides a ‘shortcut’ for successful displacement (48,56). This process allows for displacement of the incumbent strand by IN_MM_F *before* it encloses the mismatch site, and thus, its rate constant is expected to be similar to the perfectly matched input. We note that this trend for mismatch discrimination in the branch migration domain (DF = IN_MM_D > IN_MM_E > IN_MM_F) parallels what has been observed for all-DNA reactions systems (48,56,62).

Overall, these data demonstrate that the kinetics of strand displacement from PNA–DNA heteroduplexes can be controlled by mismatches within the input strand, and the relative reaction rate constants are dependent on the position of the mismatch and the stereochemical configuration of the reaction. Notably, for the sequence and toehold length examined herein, the heterochiral reaction configuration is intrinsically more sensitive to single mismatches within the toehold than its homochiral counterpart. Mismatches within the toehold are rarely employed for kinetic control of strand displacement systems (68), including nucleic acid probes designed to detect SNPs. Instead, additional activation barriers are often introduced in the form of ‘remote toehold’ (68) or ‘toehold exchange’ strategies (69) to improve mismatch discrimination. Thus, our findings expand the design capabilities for kinetic control over strand displacement. Importantly, we expect that the improved mismatch discrimination observed for heterochiral strand displacement will confer high selectivity onto corresponding heterochiral DNA devices and probes—selectivity that can be further reinforced through the data provided herein.

### Toehold stability is dependent on the reaction configuration

**Figure 4.**
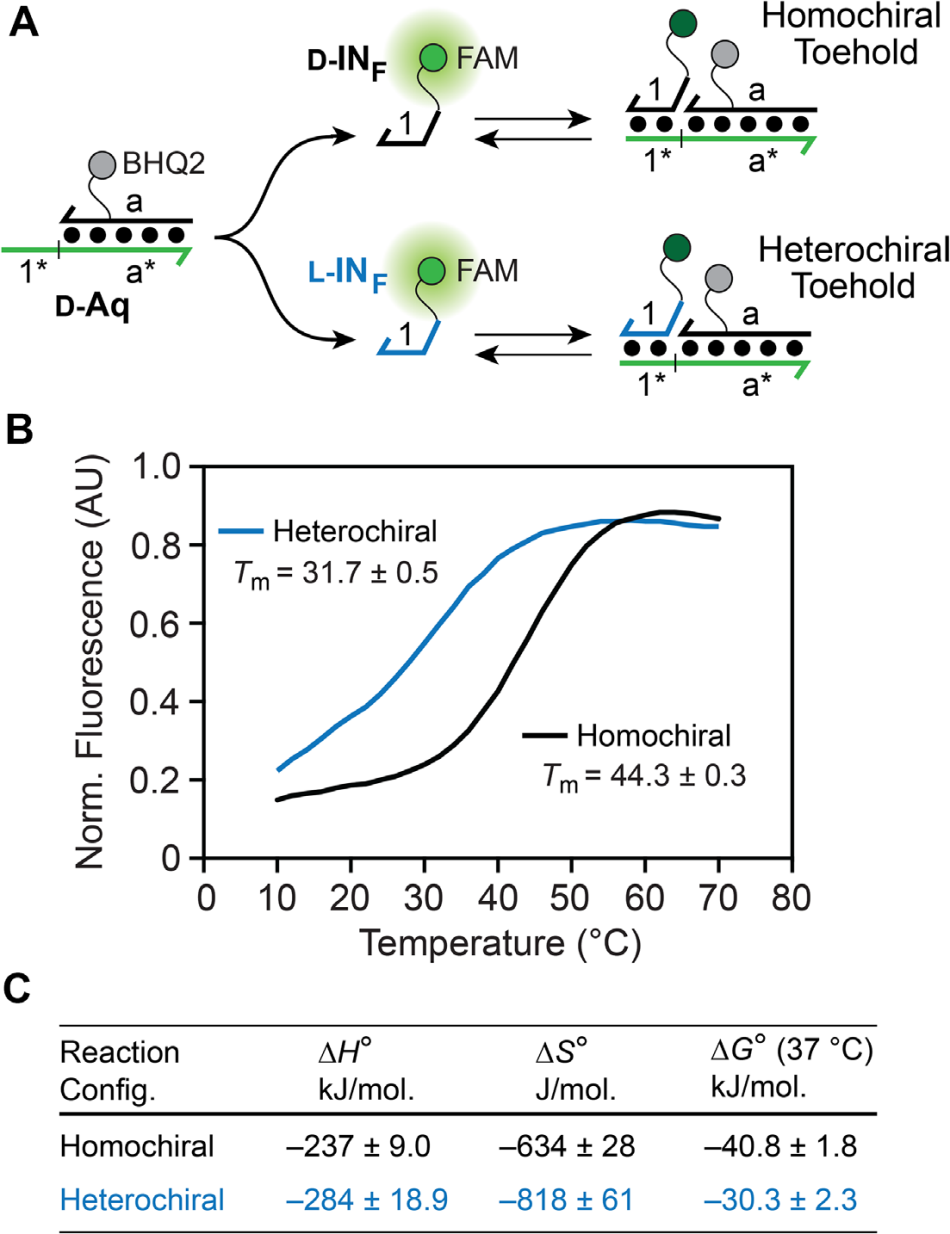
Characterization of homo- and heterochiral toehold interactions. (**A**) The model system used to monitor toehold association based on fluorescence quenching. (**B**) Fluorescence melting curves for the homo- and heterochiral toehold duplexes (*C*_t_ = 2 μM). Fluorescence values were corrected (*F*_corr_) for background fluorescence and temperature-dependent effects as defined in eq 4 (see Materials and Methods). The *T*_m_ for each toehold configuration was averaged over three melting experiments. (**C**) Thermodynamic parameters for homo- and heterochiral toehold association.

In the current reaction system (Figure 2A), as well as those reported previously (43), we found that the rate of heterochiral strand displacement from PNA–DNA heteroduplexes is significantly slower than the corresponding homochiral reaction. Given that the overall reaction rate of toehold-mediated strand displacement is strongly dependent on toehold stability, we hypothesized that differences in toehold binding energies between the homo- and heterochiral reaction configurations are the major contributor to this rate disparity. To test this, we designed an experimental system that allowed us to monitor toehold binding in isolation from branch migration (Figure 4A). The input strand (d/l-IN_F_) lacks the branch migration domain found in d-IN (domains 2 and 3), instead containing five dT residues and a 5′-6-carboxyfluorescein (6-FAM) dye (Supplementary Figure S1 and Supplementary Table S1). Thus, binding of either d-IN_F_ or l-IN_F_ to the toehold of the PNA–DNA heteroduplex d-Aq results in formation of a three-strand complex that is unable to proceed forward to displacement. Importantly, the d-DNA incumbent strand within heteroduplex d-Aq is labeled internally with a quencher (BHQ2) such that binding of either d- or l-IN_F_ to the toehold domain (1*) can be measured through fluorescence quenching.

Using this model system, we first determined the melting temperature (*T*_m_) of d-IN_F_ and l-IN_F_ with d-Aq, which correspond to the homo- and heterochiral toehold configuration, respectively (Figure 4B). Remarkably, these data revealed that the *T*_m_ of the heterochiral toehold configuration (l-IN_F_ + d-Aq) was ∼12°C lower than the corresponding homochiral toehold configuration (d-IN_F_ + d-Aq) (Figure 4B), despite their only difference being stereochemistry. In order to gain further insights, we determined the thermodynamics of toehold association for each configuration based on the concentration dependence of their melting profiles (50). The reciprocal *T*_m_ was plotted against the ln *C*_t_ (total strand concentration) and fit to a linear relationship from which Δ*H*°, Δ*S*° and Δ*G*° were derived according to established methods (Supplementary Figure S4). As observed previously (70), formation of PNA–DNA toehold complex was accompanied by large enthalpy gains and entropy losses, in agreement with the formation of a more rigid duplex structure (Figure 4C). Notably, formation of the heterochiral toehold was associated with much greater entropy losses as compared to the homochiral toehold.

Together, these data clearly show that thermal stability of the toehold duplex (e.g. the duplex formed between domains 1 and 1* on d/l-IN and d-Ai, respectively) is highly dependent on the toehold configuration, with the heterochiral toehold forming a far less stable complex with the input strand than in the homochiral toehold. Given the direct relationship between toehold stability and the rate of strand displacement (46), these results explain, in part, why heterochiral strand displacement from PNA–DNA heteroduplexes is generally slower than the corresponding homochiral reaction, and confirm toehold stability as a major contributor. This behavior may be rationalized according to the following considerations. Although PNA is achiral, upon hybridization to a chiral strand of d-DNA or l-DNA, the PNA will assume a right-handed or left-handed helical conformation, respectively (45,71). In the case of the left-handed PNA–DNA heteroduplex l-Ai, the induced left-handedness in the PNA will propagate into the single-stranded toehold domain through base-stacking interactions (72,73). This gives rise to a ‘chiral conflict’ in which the right-handed input strand (d-IN) and left-handed PNA toehold domain in l-Ai are unfavorably pre-organized for binding. Consistently, our thermodynamic data indicates that the less-favorable energy for heterochiral toehold association stems predominantly from the entropic term, which is expected for the highly ordered transition state that would accompany helical inversion of the PNA strand. This is also in agreement with prior studies showing that PNAs with an induced left-handedness bind to native d-DNA more weakly than PNAs with induced right-handedness, which is attributed to the structural organization of the PNA (entropy effects) (70,73,74). In the context of the strand displacement reactions herein, these effects are manifest through slower reaction kinetics in the heterochiral configuration.

### Strand displacement from PNA–DNA heteroduplexes is compatible with toehold exchange

**Figure 5.**
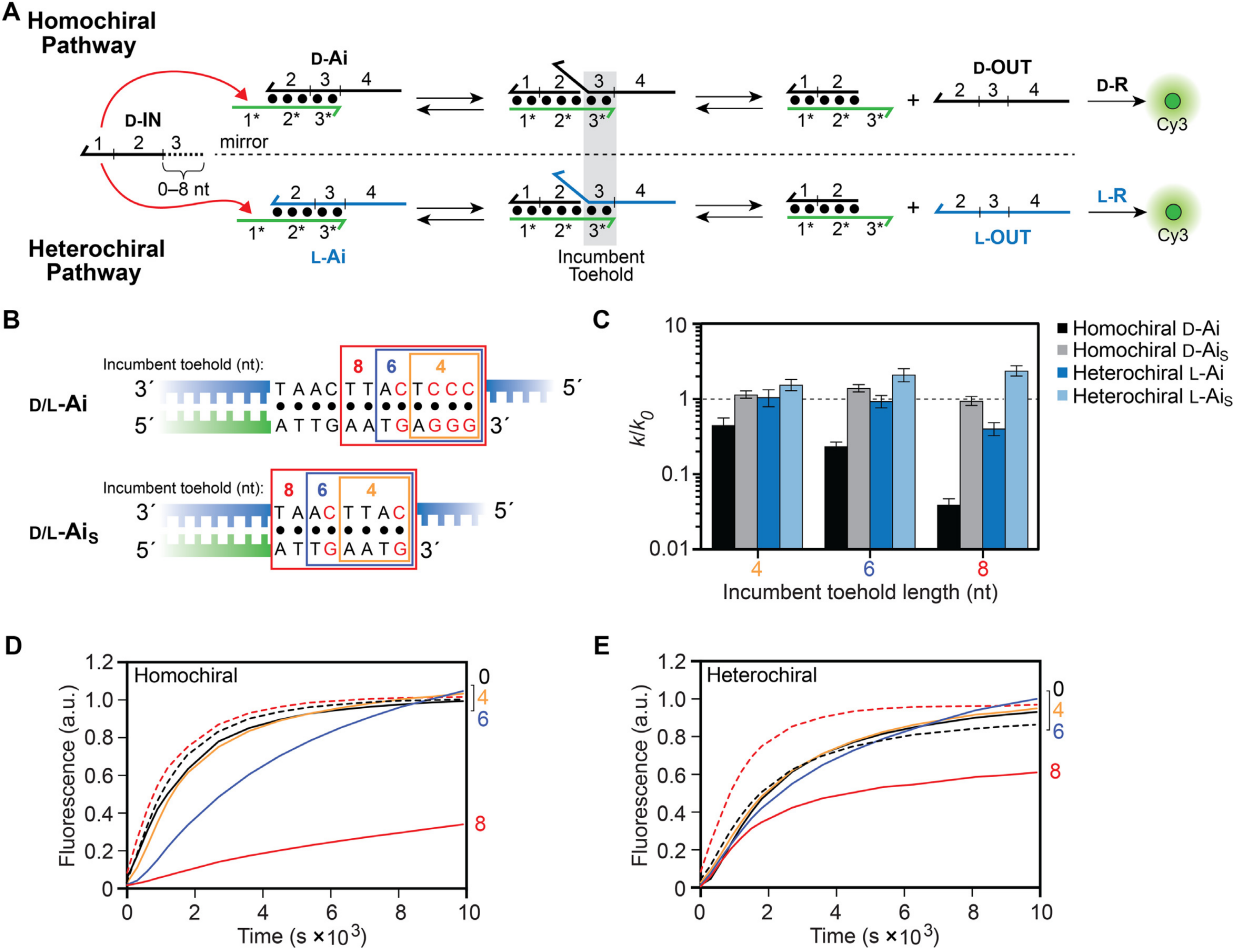
The length and nucleotide composition of the incumbent toehold affects toehold exchange on PNA–DNA heteroduplexes. (**A**) Schematic illustration of the toehold exchange mechanism for both the homo- and heterochiral reaction pathways. (**B**) Sequences of the incumbent toehold domains within d/l-Ai and its truncated version d/l-Ai_S_. Individual incumbent toeholds are boxed based on their length and red letters emphasize G/C base pairs. Incumbent toeholds are produced by truncating the input strand by the corresponding length. (**C**) Calculated rate constant as a function of incumbent toehold length (*k*) relative to the full-length input (*k*_0_) having no incumbent toehold. Error bars represent standard deviation from three independent experiments. (D, E) Fluorescence monitoring (Cy3) of toehold exchange for the homochiral (**D**) and heterochiral (**E**) reaction pathways. The length of the incumbent toehold is indicated on the right y-axis. Dotted lines indicate reactions carried out with the truncated PNA–DNA heteroduplex (Ai_S_) for the indicated incumbent toehold lengths (black = 0-nt; red = 8-nt). Reactions depicted here were carried out as described in Figure 2.

We sought to evaluate the compatibility of strand displacement from PNA–DNA heteroduplexes with the ‘toehold exchange’ mechanism (Figure 5A) (15). In this type of reaction, a truncated input strand having an incomplete branch migration domain carries out only partial displacement of the incumbent strand. The remaining base pairs, referred to as the incumbent toehold (domains 3/3* in Figure 5A), must then spontaneously dissociate for the reaction to complete. This mechanism has been evaluated extensively in the context of all-DNA strand displacement reactions (46) and provides improved control over strand displacement kinetics. The toehold exchange mechanism can also be exploited for catalysis (48,60). In presence of a fuel strand that can react with the incumbent toehold, the input strand can be regenerated over multiple turnovers. We envision that similar catalytic designs could be emulated using PNA–DNA heteroduplexes in order to develop heterochiral strand displacement devices and probes capable of signal amplification. Moreover, because the sequence of the incumbent toehold domain is independent of the input strand sequence, this approach may allow for construction of universal downstream reaction components (i.e. a modular design).

In order to demonstrate the potential for such designs using PNA–DNA heteroduplexes, we examined the rate of strand displacement initiated with versions of input d-IN_TH_8 (Figure 3A) that had been truncated by 4, 6 and 8 nucleotides from their 5′ ends, resulting in incumbent toeholds (3*) of corresponding length (Figure 5A, B). A four base pair incumbent toehold had little effect on the rate of either homo- or heterochiral reaction pathways relative to no incumbent toehold (i.e. a full-length input strand) (Figure 5C–E). This suggests that spontaneous detachment of the incumbent strand (d/l-OUT) likely occurs before the input makes significant contacts with the final four PNA–DNA base pairs of the heteroduplex (d/l-Ai). Increasing the length of the incumbent toehold further led to a decrease in strand displacement kinetics for both reaction configurations, especially for the 8 base pair incumbent toehold, which is the same length as the input toehold (1*). This observation is consistent with model studies carried out on all-DNA toehold exchange reactions: As the length of the incumbent toehold (3*) approaches that of the input toehold (1*), and the relative binding energies of both toeholds become similar, the probability of the input strand displacing the incumbent strand decreases (along with the rate) (46). Interestingly, the heterochiral reaction pathway was far less sensitive to the 8 bp incumbent toehold than its homochiral equivalent, with rate constants for strand displacement decreasing by 30-fold and 2.5-fold for the homo- and heterochiral reactions, respectively, relative to no incumbent toehold (Figure 5C). We attribute this result to weakened toehold-binding interactions for the heterochiral reaction configuration, which also extends to the incumbent toehold. Destabilization of the incumbent toehold is expected to promote displacement of the incumbent strand (l-OUT) and impede its reassociation with the incumbent toehold following displacement (i.e. the reverse reaction), together leading to overall faster forward displacement kinetics.

Given the inverse relationship between strand displacement rate constants and the stability of the incumbent toehold (46), we hypothesized that the rate could be accelerated by reducing the G/C content within an incumbent toehold without shortening it. To test this, we truncated the branch migration domain of PNA–DNA heteroduplex d/l-Ai by four base pairs, resulting in a new, shorter PNA–DNA heteroduplex (d/l-Ai_S_) having two less G/C base pairs within each of the corresponding incumbent toehold domains (3/3*) (Figure 5B). Consistent with our hypothesis, the rate of strand displacement from d/l-Ai_S_ for all incumbent toehold lengths (4, 6 and 8 nt) was at least as fast as the reaction in the absence of the incumbent toehold (Figure 5C). Notably, for the eight base pair incumbent toehold, the rate of the heterochiral reaction actually increased by ∼2-fold as a result of the reduced G/C content. Because our approach for decreasing G/C content within the incumbent toehold also shortens the branch migration domain of the PNA–DNA heteroduplex, we sought to demonstrate that this truncation does not play a substantial role in the observed rates of toehold exchange. We calculated the ratio of rate constants for strand displacement from the short (d/l-Ai_S_) and long (d/l-Ai) heteroduplexes for each of the given inputs (*k*_short_/*k*_long_, Supplementary Figure S5). For the full-length input strand (d-IN_TH_8), the ratios of *k*_short_/*k*_long_ are close to one for both reaction configurations, indicating that the rate of strand displacement is similar for both branch migration domain lengths. However, as the length of the incumbent toehold increases, *k*_short_/*k*_long_ ratios become much larger than one (Supplementary Figure S5). This strongly suggests that the sequence content of the incumbent toehold, not the length of the branch migration domain, is the primary contributor to the increased rate of toehold exchange observed for the shorter heteroduplex (d/l-Ai_S_). Thus, in addition to their relative lengths, the nucleotide content of the input and incumbent toeholds represent a key design parameter for kinetic control over toehold exchange on PNA–DNA heteroduplexes.

### RNA inputs accelerate the rate of strand displacement from PNA–DNA heteroduplexes

Detection of nucleic acid biomarkers, and in particular RNA, has widespread applications in research and medicine (75,76). Dynamic nucleic acid devices based on DNA strand displacement have been previously repurposed for sensing RNA *in vitro* and in live cells, providing a foundation for future application in bio-imaging and disease diagnosis (22,23,77). However, the kinetics of strand displacement using RNA remains relatively unexplored compared to DNA (78). In the previous sections, we have enumerated on how strand displacement from PNA–DNA heteroduplexes can have markedly different kinetic properties from previously studied all-DNA reaction systems (46,52). Therefore, it was imperative that we characterize strand displacement from PNA–DNA heteroduplexes using RNA inputs in order to establish design principles suitable for RNA detection and analysis under physiological conditions. The heterochiral reaction pathway is of particular interest in this regard because it provides the critical interface between endogenous RNA biomarkers (e.g. mRNA, microRNAs, viral RNAs, etc.) and molecular devises constructed from robust l-DNA.

**Figure 6.**
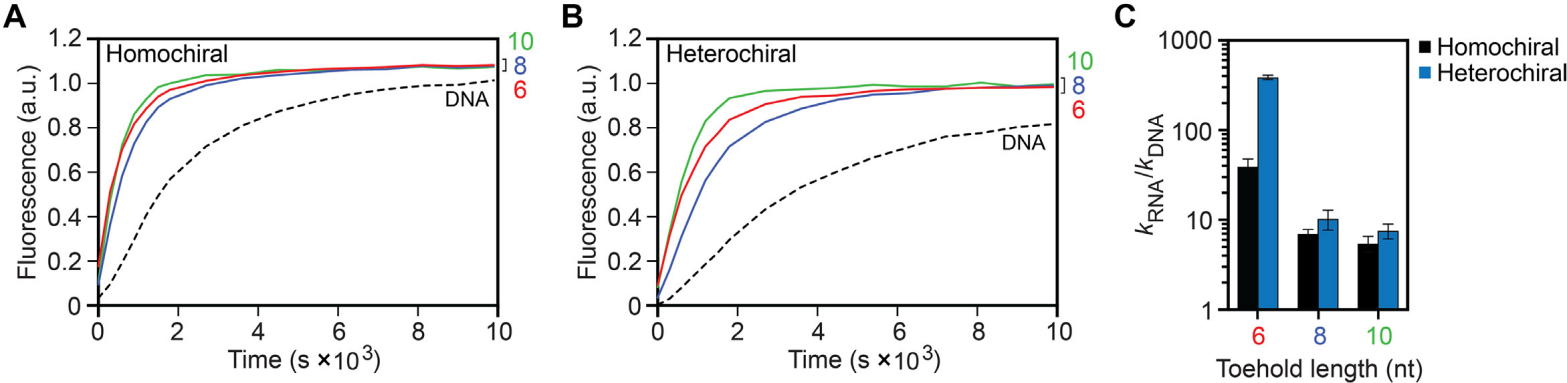
Strand displacement from PNA–DNA heteroduplexes is faster for RNA inputs than DNA inputs. (**A**, **B**) Fluorescence monitoring (Cy3) of the homochiral (A) and heterochiral (B) reaction pathways initiated with inputs RNA inputs (d-IN_RNA_) having toehold domains varying in length from 6–10 nucleotides. The length of the toehold is indicated on the right y-axis. For reference, the reaction initiated with a DNA input having a 10-nucleotide toehold is shown as a black dotted line. The reactions depicted contained 30 nM d-IN, 20 nM d/l-Ai, 60 nM d/l-R, 300 mM NaCl, 1 mM EDTA and 10 mM Tris (pH 7.6) and were carried out at 37°C. (**C**) Calculated rate constant for RNA inputs as a function of toehold length (*k*_RNA_) relative to the DNA input (*k*_DNA_) having the same length toehold. Error bars represent standard deviation from three independent experiments.

We first examined the effects of toehold length on strand-displacement using RNA versions of d-IN (d-IN_RNA_) with toehold lengths varying from 6 to 10 nucleotides (Figure 6A, B). As before, all reactions were carried out under simulated physiological conditions. For both homo- and heterochiral reaction configurations, strand displacement involving RNA inputs was drastically faster than their DNA counterparts (Figure 6C). Taking the 6-nucleotide toehold as an example, strand displacement rate constants for the RNA input were more than an order of magnitude faster than the DNA input for both reaction configurations. This was not completely unexpected given the increased stability and faster hybridization kinetics of PNA–RNA duplexes compared to PNA–DNA duplexes (58). Nevertheless, as previously observed for DNA inputs, the heterochiral reaction pathway using RNA inputs is slower than the homochiral reaction pathway for all toehold lengths tested (Supplementary Table S2). Interestingly, for both reaction configurations, the rate constant was greater for the 6-nucleotide RNA toehold than for the 8-nucleotide RNA toehold. Further examination of the RNA input revealed that is it capable of folding into a hairpin structure (Supplementary Figure S6), which is more stable for inputs with an 8- and 10-nucleotide toehold domain compared to the input with the 6-nucleotide toehold. Thus, the reduced rate of strand displacement for the RNA input with the 8-nucleotide toehold relative to the 6-nucleotide is likely due to the additional kinetic barrier of unfolding this RNA structure in some fraction of the input strand population. In the case of the 10-nucleotide toehold, the overall longer toehold domain appears to compensate for the presence of secondary structure, resulting in fast kinetics. In the context of RNA detection, this result not only demonstrates the ability of heterochiral strand displacement systems to overcome secondary structures within RNA targets, it also suggests that, through careful design, such systems may be capable of discriminating between different RNA structural conformations.

**Figure 7.**
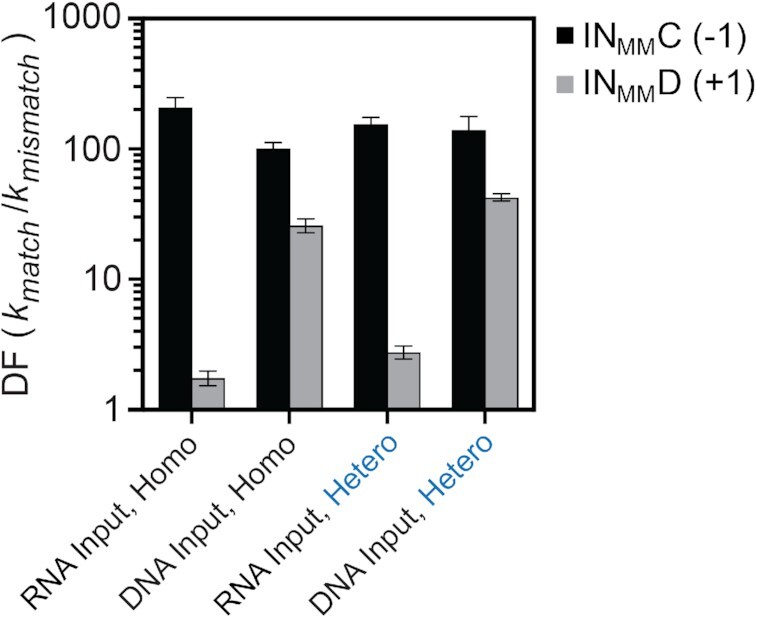
Discrimination factors (DF = *k*_match_/*k*_mismatch_) of PNA–DNA heteroduplexes towards RNA inputs having different mismatches, where *k*_match_ and *k*_mismatch_ are the calculated rate constants for a fully matched and mismatched RNA input, respectively. Error bars represent standard deviation from three independent experiments. Hetero and Homo refer to the heterochiral and homochiral reactions pathways, respectively. See Figure 3A for mismatch identity and position.

We also investigated the effect of mismatches between an RNA input strand and the PNA–DNA heteroduplex, focusing on mismatches positioned at the junction between the toehold and branch migration domains (i.e. positions −1 and +1). Mismatches at these two positions resulted in the greatest impact on strand displacement kinetics using DNA inputs, especially for the heterochiral reaction configuration. In contrast to the DNA input with a mismatch at position +1 (d-IN_MM_D), the RNA input with a mismatch at this position only modestly reduced the rate of strand displacement relative to the fully-matched input for both reaction configurations (Figure 7). Thus, the stronger toehold binding interaction of the RNA input is potentially able to compensate for the increased activation energy associated with a mismatch positioned immediately adjacent to the toehold. However, the mismatch at position −1 (d-IN_MM_C) within the toehold domain of the RNA input retains its strong inhibitory effect, decreasing the rate of strand displacement by at least two orders of magnitude relative to the fully-matched RNA input (Figure 7). Thus, a mismatch at this position (−1) will be useful for designing kinetic probes capable of discriminating between RNAs based on SNPs.

Together, these studies demonstrate that strand displacement rates for PNA–DNA heteroduplexes are substantially enhanced, by up to two orders of magnitude, upon substitution of RNA inputs for DNA inputs. Furthermore, high sensitivity to single-nucleotide mismatches, when appropriately positioned, can be maintained. With regard to heterochiral strand displacement, the increased reaction kinetics using RNA inputs will greatly benefit future applications aimed at interfacing endogenous RNAs with molecular devices and sensors constructed from biostable l-DNA. Moreover, the potential sensitivity of heterochiral strand displacement to RNA secondary structures is interesting and deserves to be explored further. We anticipate that discrimination between RNA structural conformations could be exploited in a broad range of applications beyond primary sequence detection.

## CONCLUSIONS

Through detailed experimental analysis, we have demonstrated that several common design parameters for controlling DNA strand displacement kinetics, including toehold length and mismatches, can also be applied to strand displacement from PNA–DNA heteroduplexes. Although important differences exist, we found that the rate of PNA–DNA strand displacement reactions can be tuned in a manner that is mostly analogous to traditional all-DNA reactions. For example, reaction rates increase proportionally with toehold length and mismatches positioned proximal to the toehold domain strongly inhibit strand displacement. These similarities suggest that strand displacement systems based on PNA–DNA heteroduplexes can be predictably engineered to undergo similar dynamic behaviors as those constructed solely from DNA. Indeed, we showed that PNA–DNA heteroduplexes are compatible with toehold exchange. Importantly, our careful parameterization of the heterochiral strand displacement pathway, which serves as the key interface between l-DNA and endogenous d-nucleic acids, provides an important contribution to the rational design and optimization of dynamic l-DNA-based circuits and nanodevices capable of interfacing with biological systems.

In addition to their similarities, we find that strand displacement reactions from PNA–DNA heteroduplexes exhibit several unique, and potentially advantageous, characteristics relative to their all-DNA counterparts. In particular, we show that stereochemistry, a parameter unique to our system, offers an additional layer of kinetic control not possible using conventional all-DNA strand displacement. We anticipate that this capability will greatly expand the types of dynamic behaviors that can be programmed into nucleic acid-based devices. For example, one could imagine building a kinetic ‘thresholding’ gate based on the rate discrepancy between identical homochiral and heterochiral strand displacement reactions as a straightforward alternative to previous designs based on toehold length and/or composition (12). Toehold exchange reactions using PNA–DNA heteroduplexes also exhibit unique kinetic behaviors, especially for the heterochiral reaction configuration. We show that heterochiral toehold exchange is mostly insensitive to the length of the incumbent toehold (3*), even as its length approaches that of the input toehold domain (1*). This is in direct contrast to the expected trends for introducing incumbent toeholds into all-DNA systems (46), and could be exploited to develop heterochiral strand displacement devices and probes capable of rapid signal amplification.

During the course of this work, we explored the underlying cause of the observed rate discrepancies between homo- and heterochiral strand displacement reactions, focusing on the stability of the corresponding toehold domains. Melting temperature analysis revealed that the toehold domain in the heterochiral reaction configuration forms a far less stable complex with the input strand than does the toehold domain in the homochiral reaction configuration. Thermodynamic data indicated that the less-favorable free energy for heterochiral toehold association stems predominantly from the entropic term, which we attribute to unfavorable pre-organization between the induced left-handed PNA toehold and right-handed input strand. This observation not only provides important insights into the biophysical mechanisms of heterochiral strand displacement, but also into the broader role of molecular organization in controlling strand displacement kinetics, which could be exploited elsewhere as a tool for modulating reaction rates. For example, our results suggest that the rate of heterochiral strand displacement could be increased by enforcing a right-handed helical conformation on the toehold domain, possibly by using *chiral* PNA monomers such as those containing modifications at the γ-position of the backbone (73). It is also worth noting that this study provides further experimental evidence supporting the extended propagation of induced helicity through single-stranded, achiral PNA.

Most importantly, heterochiral strand displacement reactions exhibited fast reaction kinetics with RNA inputs and maintained a high level of mismatch discrimination when appropriately positioned. This further demonstrates the potential applications of this technology for the detection and manipulation of biologically relevant RNA molecules. Indeed, we have previously shown heterochiral strand displacement circuits composed of l-DNA/RNA greatly outperform their all-DNA counterparts in living cells and can be directly interfaced with endogenous RNAs (45,79). We are continuing to pursue routes to increase the performance of heterochiral strand displacement systems, both *in vitro* and *in vivo*. This work now provides a more solid foundation from which to base future designs. For example, we found that mismatch discrimination is dependent on whether the input strand was composed of DNA or RNA (Figure 7). This not only highlights the importance of studying both types of inputs, which is seldomly done, but also provides valuable information for engineering heterochiral strand displacement probes with increased selectivity for RNA based on SNPs. It should be mentioned that the rate of heterochiral strand displacement, especially for RNA inputs, may vary significantly depending on the sequence (and secondary structure), and it will be important to further characterize these effects in the future.

Overall, this work establishes a basic set of design considerations to guide future development of strand displacement systems based on the unique properties of PNA–DNA heteroduplexes. In particular, we expect that the detailed characterization of heterochiral strand displacement kinetics provided herein, along with the increasing availability of l-oligonucleotides, will broaden the scope and applicability of l-DNA/RNA-based circuits and other nanodevices for practical applications at the interface with biology.

## DATA AVAILABILITY

The data generated during all experiments is available from the author upon reasonable request.

## Supplementary Material

gkab499_Supplemental_File

## Notes

Present address: Brian E. Young, Department of Radiology, Stanford University, Stanford, CA 94305, USA.
