# Supplementary material for: Kinetics of heterochiral strand displacement from PNA–DNA heteroduplexes
Source: Nucleic Acids Res. 2021 Jun 14;49(11):6114–27. doi: 10.1093/nar/gkab499 (PMC8216467; doi:10.1093/nar/gkab499)
Supplement: gkab499_Supplemental_File [file gkab499_supplemental_file.pdf]

Supporting Information

Kinetics of Heterochiral Strand Displacement From PNA-DNA Heteroduplexes

Nandini Kundu[1,†], Brian E. Young[1,†,‡], and Jonathan T. Sczepanski[1,*]

[1] Department of Chemistry, Texas A&M University, College Station, Texas, 77843, USA

[†]These authors contributed equally to this work

[‡] Current address: Department of Radiology, Stanford University, Stanford, California, 94305, USA

I. Supplementary Figures.

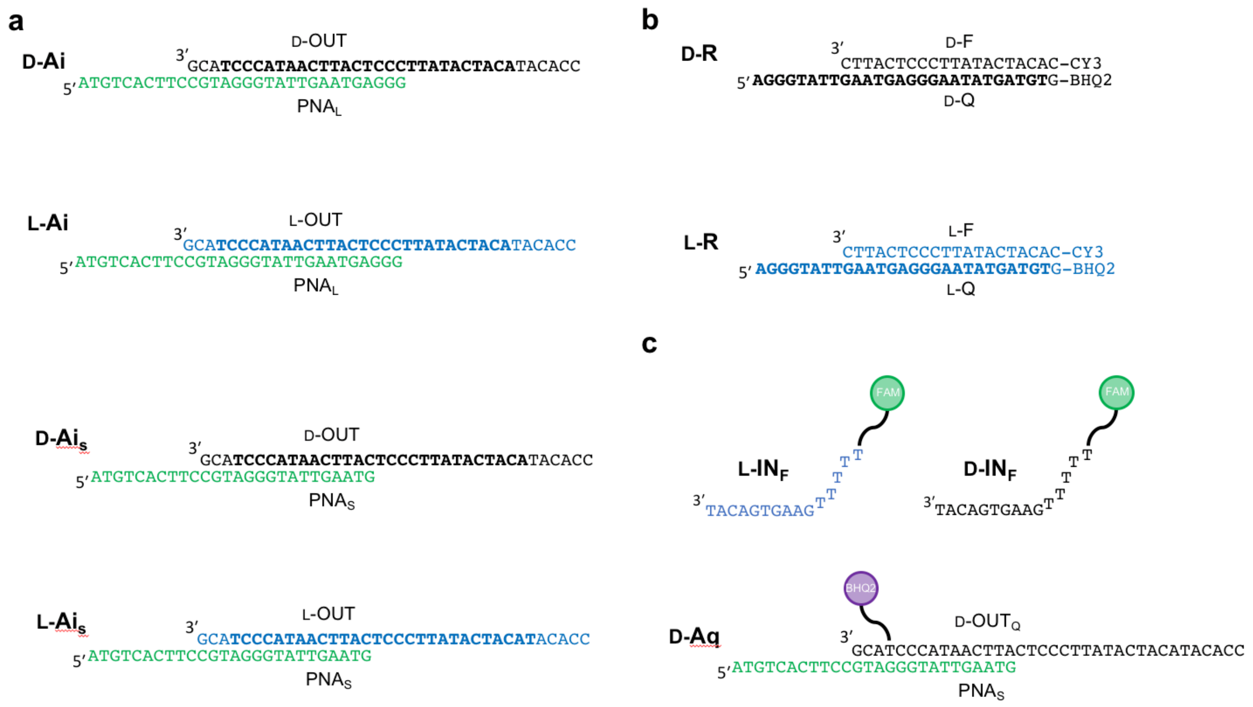

Figure S1. (a) Sequences of PNA-DNA heteroduplexes D/L-Ai and D/L-Ai$_S$. (b) Sequences of reporter complexes D-R and L-R. Sequence complementarity between D/L-OUT and D/L-Q has been emphasized using bold text. (c) Sequences of D-IN$_F$, L-IN$_F$ and heteroduplex D-Aq used in the toehold melting experiments.

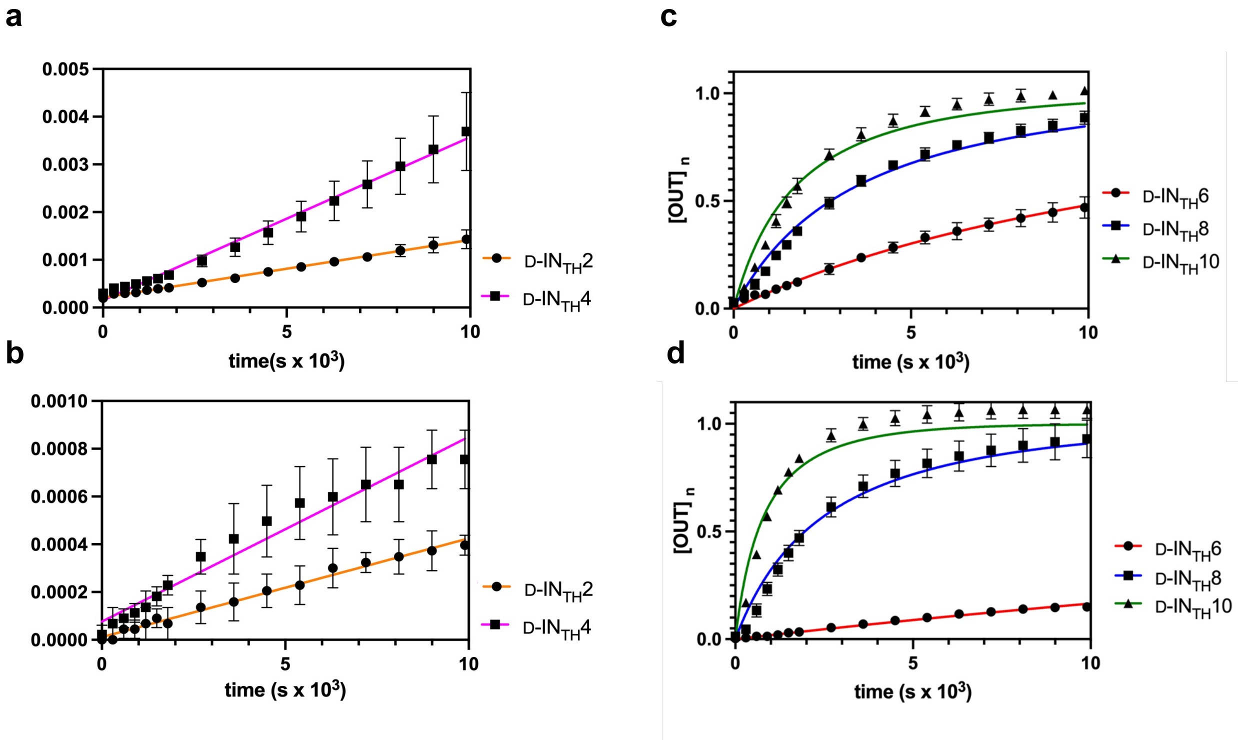

Figure S2. Representative sets of data and fits used to derive second-order rate constants for homo- and heterochiral strand displacement reactions with different toehold lengths (See Materials and Methods for details). (a,b) Representative linear fits for slow homochiral (a) and heterochiral (b) strand displacement reactions with inputs containing 2 or 4 nucleotide toeholds (D-IN$_{TH}$2 and D-IN$_{TH}$4, respectively). The left-hand side of equation 2 (Materials and Methods) was plotted on the y-axis against time. Rate constants (Table S2) were extracted from the slope of the fit line. (c,d) Representative fits for fast homochiral (c) and heterochiral (d) strand displacement reactions with inputs containing 6, 8 and 10 nucleotide toeholds (D-IN$_{TH}$6, D-IN$_{TH}$8 and D-IN$_{TH}$10, respectively). Rate constants (Table S2) were extracted by fitting equation 3 (Materials and Methods) to all the data points.

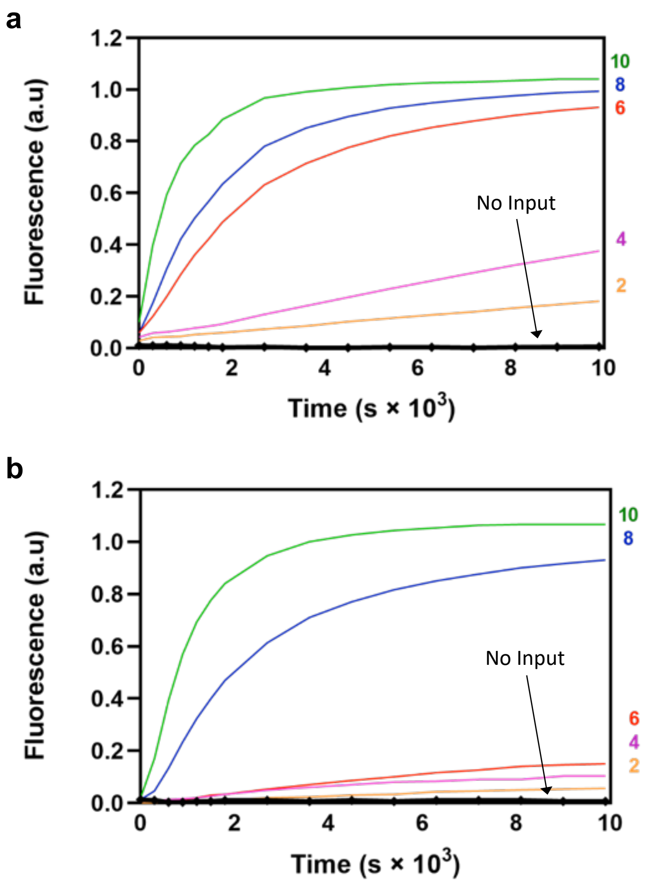

Figure S3. Strand displacement from a PNA-DNA heteroduplex is strongly dependent on toehold length. Under the reaction conditions used in this work, essentially no fluorescence signal was observed in the absence of an input for either the homochiral (a) or heterochiral (b) reactions. The length of the toehold is indicated on the right y-axis. The depicted reactions contained either 0 nM or 150 nM input strand, 100 nM D/L-Ai, 300 nM D/L-R, 300 mM NaCl, 1 mM EDTA, and 10 mM Tris (pH 7.6) and were carried out at 37 °C.

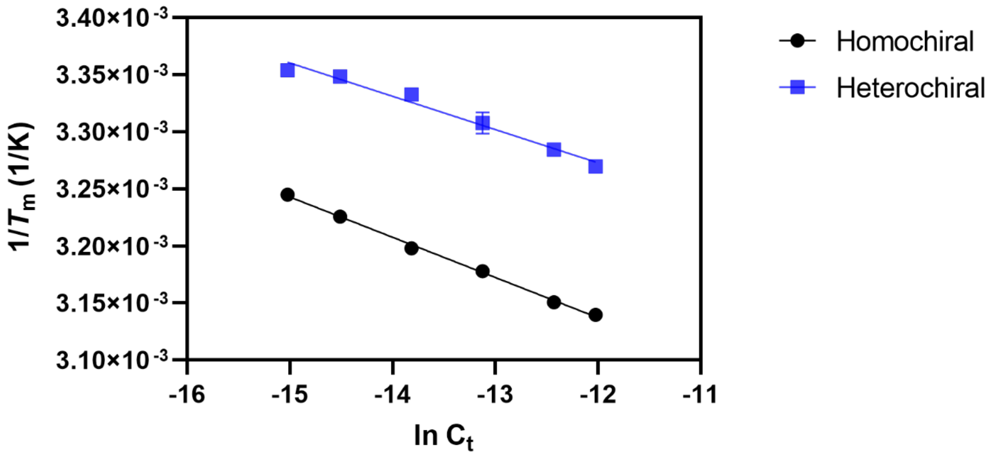

Figure S4. Thermodynamic parameters ΔH° and ΔS° were extracted from the linear best fit line of either the reciprocal melting temperature ($1/T_m$) of the homochiral or heterochiral melting data against ln C_t, where C_t is the total component concentration (strand D/L-IN_F + D-Aq) used in each experiment.

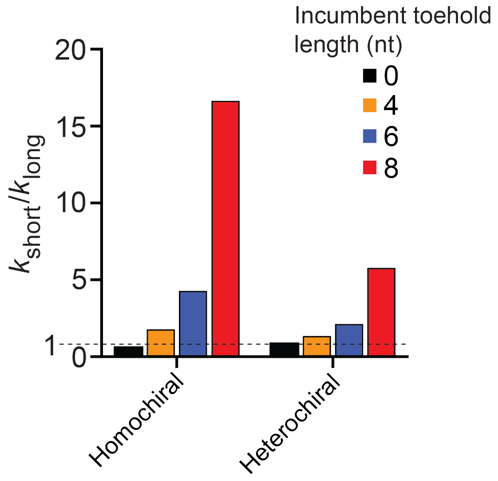

Figure S5. Ratio of rate constants for strand displacement from the short (D/L-Ai$_S$) and long (D/L-Ai) heteroduplexes for each of the given incumbent toehold lengths.

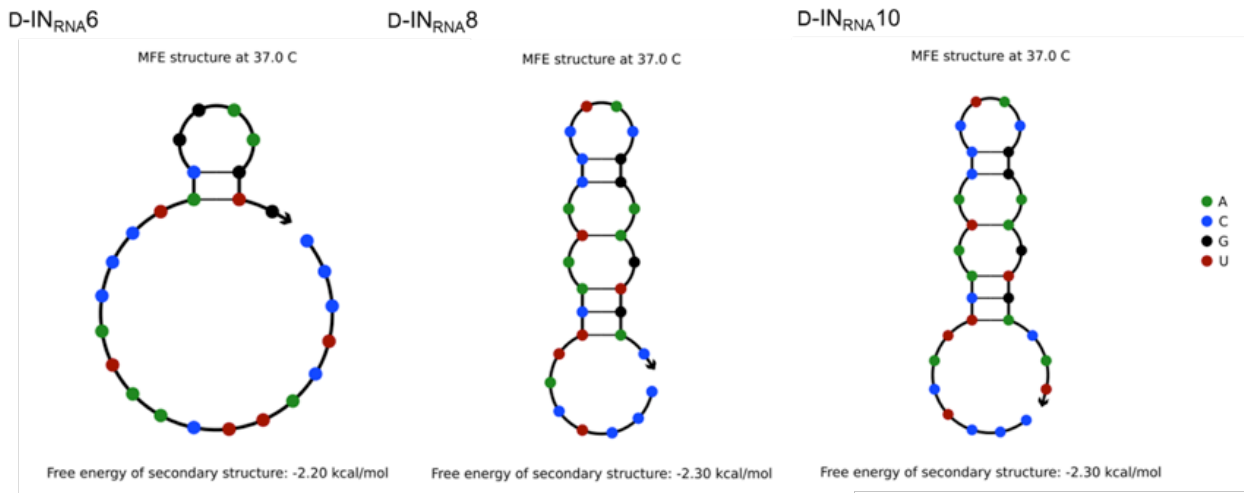

Figure S6. RNA inputs with 8 and 10 nucleotide toeholds (IN$_{RNA}$8 and IN$_{RNA}$10, respectively) are predicted to form more extensive secondary structure than the 6 toehold RNA input (IN$_{RNA}$6). See Table S1 for sequences. RNA secondary structure was predicted using NUPACK.

II. Supplementary Tables.

Table S1. Names, sequences, and chirality of strands used in this work. Dye and quencher modifications are indicated in bold, while mismatches within indicated strands are italicized. D-DNA (black), L-DNA (blue), PNA (green), and D-RNA (red) are indicated by color.

Sequence Name	Sequence Identity 5'→3'	Oligomer Stereochemistry
PNA_L	ATGTCACTTCCGTAGGGTATTGAATGAGGG	Achiral
PNA_S	ATGTCACTTCCGTAGGGTATTGAATG	Achiral
D-OUT	CCACATACATCATATTCCCTCATTCAATACCCTACG	D
D-F	**Cy3**-CACATCATATTCCCTCATTC	D
D-Q	AGGGTATTGAATGAGGGAATATGATGTG-**BHQ2**	D
D-IN$_{TH}$10	CCCTCATTCAATACCCTACGGAAGTGACAT	D
D-IN$_{TH}$8	CCCTCATTCAATACCCTACGGAAGTGAC	D
D-IN$_{TH}$6	CCCTCATTCAATACCCTACGGAAGTG	D
D-IN$_{TH}$4	CCCTCATTCAATACCCTACGGAAG	D
D-IN$_{TH}$2	CCCTCATTCAATACCCTACGGA	D
D-IN$_{MM}$A	CCCTCATTCAATACCCTACGGAAG*A*GAC	D
D-IN$_{MM}$B	CCCTCATTCAATACCCTACGGAA*CA*GAC	D
D-IN$_{MM}$C	CCCTCATTCAATACCCTACG*C*AACTGAC	D
D-IN$_{MM}$D	CCCTCATTCAATACCCTAC*C*GAAGTGAC	D
D-IN$_{MM}$E	CCCTCATTCA*T*TACCCTACGGAAGTGAC	D
D-IN$_{MM}$F	CC*G*TCATTCAATACCCTACGGAAGTGAC	D
D-IN$_{INC}$8	CAATACCCTACGGAAGTGAC	D
D-IN$_{INC}$6	TTCAATACCCTACGGAAGTGAC	D
D-IN$_{INC}$4	CATTCAATACCCTACGGAAGTGAC	D
D-IN$_{INC}$8(S)	ACCCTACGGAAGTGAC	D
D-IN$_{INC}$6(S)	ATACCCTACGGAAGTGAC	D
D-IN$_{INC}$4(S)	CAATACCCTACGGAAGTGAC	D
D-IN$_{RNA}$10	CCCUCAUUCAAUACCCUACGGAAGUGACAU	D
D-IN$_{RNA}$ 8	CCCUCAUUCAAUACCCUACGGAAGUGAC	D
D-IN$_{RNA}$ 6	CCCUCAUUCAAUACCCUACGGAAGUG	D
D-IN$_{RNA}$C	CCCUCAUUCAAUACCCUACG*C*AACUGAC	D
D-IN$_{RNA}$D	CCCUCAUUCAAUACCCUAC*C*GAAGUGAC	D
D-IN$_F$	**FAM**-TTTTTGAAGTGACAT	D
D-OUT$_q$	CCACATACATCATATTCCCTCATTCAATACCC[T(**BHQ2**)] ACG	D
L-OUT	CCACATACATCATATTCCCTCATTCAATACCCTACG	L
L-F	**Cy3**-CACATCATATTCCCTCATTC	L
L-Q	AGGGTATTGAATGAGGGAATATGATGTG-**BHQ2**	L
L-IN$_F$	**FAM**-TTTTTGAAGTGACAT	L
L-IN$_{TH}$10	CCCTCATTCAATACCCTACGGAAGTGACAT	L
L-IN$_{RNA}$10	CCCUCAUUCAAUACCCUACGGAAGUGACAU	L

Table S2. Calculated rate constants for homochiral and heterochiral reactions described in the text. Rates represent the average of 3 fittings and their standard deviation.

Input	Description	Heterochiral *k* ($M^{-1}s^{-1}$)	Homochiral *k* ($M^{-1}s^{-1}$)
D-IN$_{TH}$10	10 nt toehold	9194 ± 399.34[b]	20953.33 ± 1597.55[b]
D-IN$_{TH}$8	8 nt toehold	2971.33 ± 474.71[b]	10546 ± 720.11[b]
D-IN$_{TH}$6	6 nt toehold	127.30 ± 1.39[b]	2704.67 ± 310.84[a]
D-IN$_{TH}$4	4 nt toehold	88.97 ± 15.69[a]	365.23 ± 57.99[a]
D-IN$_{TH}$2	2 nt toehold	43.09 ± 7.67[a]	152.70 ± 11.91[a]
D-IN$_{TH}$0	0 nt toehold	7 ± 1.28[a]	37.97 ± 1.80[a]
D-IN$_{MM}$A	Mismatched input A	100.99 ± 16.29[a]	1810 ± 338.91[a]
D-IN$_{MM}$B	Mismatched input B	82.22 ± 6.29[a]	505.93 ± 77.08[a]
D-IN$_{MM}$C	Mismatched input C	22.43 ± 5.41[a]	105.37 ± 9.18[a]
D-IN$_{MM}$D	Mismatched input D	69.90 ± 3.56[a]	411.13 ± 41.99[a]
D-IN$_{MM}$E	Mismatched input E	1837 ± 405.33[b]	4348.33 ± 426.92[b]
D-IN$_{MM}$F	Mismatched input F	3507 ± 425.97[b]	5866 ± 90.71[b]
D-IN$_{INC}$8	8 nt incumbent toehold on D/L-Ai	1208.33 ± 43.44[b]	417 ± 35.25[b]
D-IN$_{INC}$6	6 nt incumbent toehold on D/L-Ai	2799.67 ± 74.82[b]	2493 ± 231.67[b]
D-IN$_{INC}$4	4 nt incumbent toehold on D/L-Ai	3155.67 ± 298.27[b]	4748.67 ± 196.54[b]
D-IN$_{TH}$8 (S)	8 nt toehold on D/L-Ai$_S$	2966.33 ± 370.46[b]	7805.33±280.55[b]
D-IN$_{INC}$8(S)	8 nt incumbent toehold on D/L-Ai$_S$	7112.67 ± 221.61[b]	6989 ± 530.47[b]
D-IN$_{INC}$6(S)	6 nt incumbent toehold on D/L-Ai$_S$	6282.33 ± 454.18[b]	10967.67 ± 1271.73[b]
D-IN$_{INC}$4(S)	4 nt incumbent toehold on D/L-Ai$_S$	4618.33 ± 218.14[b]	9008.67 ± 464.88[b]
D-IN$_{RNA}$10	10 nt toehold with RNA input	69572.67 ± 9911.76[b]	114444.33 ± 13865[b]
D-IN$_{RNA}$8	8 nt toehold with RNA input	30520.67 ± 2762.89[b]	73907.67 ± 3600.21[b]
D-IN$_{RNA}$6	6 nt toehold with RNA input	49470.33 ± 2267.36[b]	106228.3 ± 10418.23[b]
D-IN$_{RNA}$C	RNA input with mismatch C	196 ± 6.24[a]	352.17 ± 47.00[a]
D-IN$_{RNA}$D	RNA input with mismatch D	11039.67 ± 267.09[b]	42165.67 ± 3370.70[b]

[a]Rate constant extracted using equation 2 as described in the Materials and Methods Section.
[b]Rate constant extracted using equation 3 as described in the Materials and Methods Section.
